# Novel methods for genotype imputation to whole-genome sequence and a simple linear model to predict imputation accuracy

**DOI:** 10.1186/s12863-017-0588-1

**Published:** 2017-12-27

**Authors:** Steven G. Larmer, Mehdi Sargolzaei, Luiz F. Brito, Ricardo V. Ventura, Flávio S. Schenkel

**Affiliations:** 1 Centre for Genetic Improvement of Livestock, Department of Animal Biosciences, University of Guelph, 50 Stone Road East, Guelph, ON N1G 2W1 Canada; 2 The Semex Alliance, 5653 Highway 6 North, Guelph, ON N1H 6J2 Canada; 3 Bringing Intelligence Opportunities, 294 Mill St. East, Elora, ON N0B 1S0 Canada

**Keywords:** Cattle genomics, Genomic clustering, Genotype imputation, Sequencing data

## Abstract

**Background:**

Accurate imputation plays a major role in genomic studies of livestock industries, where the number of genotyped or sequenced animals is limited by costs. This study explored methods to create an ideal reference population for imputation to Next Generation Sequencing data in cattle.

**Methods:**

Methods for clustering of animals for imputation were explored, using 1000 Bull Genomes Project sequence data on 1146 animals from a variety of beef and dairy breeds. Imputation from 50 K to 777 K was first carried out to choose an ideal clustering method, using ADMIXTURE or PLINK clustering algorithms with either genotypes or reconstructed haplotypes.

**Results:**

Due to efficiency, accuracy and ease of use, clustering with PLINK using haplotypes as quasi-genotypes was chosen as the most advantageous grouping method. It was found that using a clustered population slightly decreased computing time, while maintaining accuracy across the population. Although overall accuracy remained the same, a slight increase in accuracy was observed for groups of animals in some breeds (primarily purebred beef cattle from breeds with fewer sequenced animals) and for other groups, primarily crossbreed animals, a slight decrease in accuracy was observed. However, it was noted that some animals in each breed were poorly imputed across all methods. When imputed sequences were included in the reference population to aid imputation of poorly imputed animals, a small increase in overall accuracy was observed for nearly every individual in the population. Two models were created to predict imputation accuracy, a complete model using all information available including Euclidean distances from genotypes and haplotypes, pedigree information, and clustering groups and a simple model using only breed and an Euclidean distance matrix as predictors. Both models were successful in predicting imputation accuracy, with correlations between predicted and true imputation accuracy as measured by concordance rate of 0.87 and 0.83, respectively.

**Conclusions:**

A clustering methodology can be very useful to subgroup cattle for efficient genotype imputation. In addition, accuracy of genotype imputation from medium to high-density Single Nucleotide Polymorphisms (SNP) chip panels to whole-genome sequence can be predicted well using a simple linear model defined in this study.

## Background

Genotype imputation algorithms have played a crucial role on increasing the size of a population with high-density (HD) or sequence genotypes when cost is the limiting factor in studies design or for practical applications in livestock industries. Imputation in cattle from low density SNP chip panels to 50 K or 777 K genotype panels has been shown to be highly accurate, especially in breeds with large reference populations of animals with HD genotypes, and high levels of linkage disequilibrium (LD) across the genome [1, 2].

The challenges for accurate imputation to sequence in cattle is due to the high incidence of low minor allele frequency variants that are much more difficult to impute with good accuracy. However, imputation from the 777 K SNP chip panel to full sequence is possible with moderate accuracy, especially for variants above a minor allele threshold of 5% [3]. Less common variants are much more difficult to impute accurately, and any variants that appear at a frequency lower than 0.05 are imputed extremely poorly [4]. To increase the accuracy of imputation in cattle populations, an ideal group of animals need to be sequenced in order to maximize the number of haplotypes available in the reference population [5].

Another important concern to maximize imputation accuracy is to select animals for sequence that have been shown to be more genetically related to all other animals in the population, especially those that are to be imputed from lower density SNP chip panels [6]. It has been reported in the literature that a stronger average relationship between reference and imputation population will maximize imputation accuracy. However, with higher density panels for imputation, the impact of genetic relationship decreases [2]. An additional concern for maximizing accuracy of imputation is the size of the reference population. The accuracy of imputation improves as the reference population size increases, given that animals added to the reference share haplotypes with those in the imputation population. When imputing from a very low density panel in comparison to the reference, it has been shown that combining reference genotypes from various less related breeds (i.e., that do not share many haplotypes) will decrease imputation accuracy [7].

Accuracy of imputation from HD to full sequence across all breeds has been shown to be more effective than imputation carried out within a single breed [4]. However, there may be a down-side to combining all breeds together for imputation. It is possible that some breeds, especially those selected for different purposes, may be too distant to aid genotype imputation of animals in those other breeds, and could add spuriously associated haplotypes to the imputation process that would decrease overall accuracy. It has been shown that using a clustering algorithm to better group animals into accurate sub-populations could aid in the accuracy of genomic selection for multi-breed and crossbred beef cattle [8].

A large sequenced population of cattle has a wide array of applications for both association analysis and genomic selection, leading to a better understanding and ability to utilize the bovine genome for more accurate genomic selection. In humans, a large sequenced population has already been shown to be useful to identify many rare variants associated with traits of interest [9]. In cattle, it lends to the possibility of fine-mapping genes for traits of economic interest, and potentially a better understanding of the genetic architecture of traits. Having multiple breeds with sequenced populations could significantly aid the detection of rare causal variants [10]. Imputing animals that are closely related to the reference population, assumed to have a high imputation accuracy, and then imputing less related animals subsequently may be a viable method to increase accuracy. Imputing the large number of existing genotypes to sequence is a crucial step to maximize the power and accuracy of Next Generation Sequencing (NGS) studies, and all methods should be thoroughly explored.

Individual imputation accuracy may also have a significant effect on the accuracy of subsequent Genome-Wide Association Studies (GWAS) and genomic prediction steps after a population is imputed to sequence. Removing poorly imputed individuals would be advantageous to the accuracy of studies using imputed sequences, as well as routine use of sequences in genomic evaluations in the future.

In this study we investigated imputation accuracy from 50 K as well as 777 K to sequence genotypes, using clustering methodologies as well as imputing across all animals to determine the best methodology for imputation from various densities. Clustering algorithms using both genotypes and haplotypes were evaluated. In addition, we also explored a method to impute animals in a step-wise manner, imputing first the most highly related animals, based on Euclidean distances, and then including those imputed genotypes in the reference population for imputation of less related genotypes. We also investigated a predictive model for imputation accuracy based on clustering, breed, and relationship data using only low-density genotypes.

## Methods

### Data

Full genome sequences from 1146 bulls from 27 pure and composite breeds of both dairy and beef cattle were used in this study. The dataset is property of the 1000 Bull Genomes Project (Run 4.0). Bulls were sequenced at an average coverage depth of 11X. Pedigree information was available for a limited number of animals, which resulted in a relatively small degree of interconnectedness between animals in the population, even within breed, relatedness was taken into account. Based solely on pedigree information, animals averaged having 0.2 sequenced parents and 0.2 sequenced offspring. Given that for most breeds, these are the most influential sires worldwide, which are known to be highly related with one another, this is most likely an underestimation of total relatedness. However, there were measures taken in most populations to maximize the total number of sequenced haplotypes, so even limited pedigree relatedness within one generation may closely reflect the true level of relatedness of all sequenced individuals. Pedigree information was used for all further analysis, but did not make a significant impact on any results, and could have been excluded with no serious effect. The sequences contained 36,916,857 variants before data editing. Loci with extremely low minor allele frequencies (occurring less than four times across all individuals) were removed to control for false positive sequence calls. It has also been shown that these variants cannot be imputed without a much larger reference population. After removing these sites, 23,873,140 variants remained for further analysis.

### Clustering

Sequences were first filtered to only contain the SNPs present on the 50 K Illumina BovineSNP50 beadchip (Illumina Inc., San Diego, USA). All animals were filtered to contain only these 48,455 variants in common between sequence and the 50 K Illumina BovineSNP50 beadchip. This was done to mimic the scenario where 50 K genotypes are to be imputed to sequence in two steps, and clustering analysis could be performed on 50 K genotypes before imputation. Three clustering methods: 1) ADMIXTURE, 2) PLINK using genotypes, and 3) PLINK using reconstructed haplotypes were explored for imputation from 50 K to 777 K, and compared to imputation using all animals in one population. The method that provided the highest overall accuracy of imputation from 50 K to 777 K was then selected to be compared to imputation using all individuals from 777 K to sequence.

### ADMIXTURE model

The ADMIXTURE model, as detailed by Alexander et al. [11], allows for the detection of “chunks” of chromosomes that are unbroken and that originally stemmed from an ancestral population. The model was applied to each animal to determine the proportion of the genome estimated to come from each cluster for each animal. The optimal number of clusters was chosen by iterating over one to 25 clusters and minimizing the coefficient of variation error, as calculated by the ADMIXTURE v1.3 software (https://www.genetics.ucla.edu/software/admixture/, [11]). A total of four overall clusters were used for genotype imputation in this method.

### PLINK model (identical by state clustering)

Identical by state (IBS) clustering was carried out using the PLINK v1.07 software [12]. This algorithm compares pairwise IBS metrics for each pair of individuals. This IBS metric is calculated as the genetic distance between two individuals based on genotype. Each animal is then considered a cluster, and each cluster is compared, starting with clusters that have the smallest distance between them, using a set of metrics [12] to determine if these two clusters should be merged. PLINK v1.07 software also allows for a fixed number of clusters to be set. To ensure adequate population sizes for each cluster under imputation, fixed cluster sizes were tested, beginning with six, and reduced until no cluster had fewer than 150 animals. This resulted in five clusters being created for use in imputation. As imputation relies primarily on shared haplotypes (i.e. not shared genotypes) haplotypes were reconstructed using the haplotype phasing Expectation-Maximization algorithm in PLINK v1.07 software. Although phasing across breeds may be inaccurate, and could potentially lead to less accurate clustering, for small breeds it is impossible to accurately phase individuals within breed. Four SNP windows were chosen for this analysis, and haplotypes were then treated as multi-allelic SNPs for clustering. IBS clustering was carried out in the same manner as for genotypes using the PLINK software algorithm. Five clusters were once again chosen as the optimal number for imputation. There was a large concordance between clusters using genotype and haplotype information (data not shown). However many of the multi-breed and composite breed individuals were clustered differently when using haplotypes instead of genotypes.

### Genotype imputation

Imputation was carried out for each group by filtering the sequence calls for that group. For each imputation scenario, reference and imputation groups were created at random by splitting each of the scenarios into five subgroups. These five subgroups were divided randomly, within breed, and then breed subgroups were combined together at random to make the five groups for imputation. For clustering analysis, this meant a total of 20 or 25 imputation groups were used to impute every animal. Computation time was also explored for each of these subgroups, as well as time per animal to ensure this process was not limiting if it were to be implemented on larger populations in a commercial setting. Therefore, only the sites concordant with the 50 K panel were kept, filtering the remainder of the groups to SNPs contained on the Illumina 777 K Bovine Illumina HD Beadchip. This was carried out for all five groups and, consequently, each animal in the population was imputed from their mock 50 K panel genotype to a mock 777 K panel. Imputation accuracy for this step was then investigated. Imputation was carried out once again using the same groups, from imputed 777 K to the full sequence genotype for all animals as well as the highest accuracy clustering groups from imputation from 50 K to 777 K, which was clustering based on haplotypes using the PLINK algorithm. Indels were excluded for the imputation analysis. All the imputation were performed using the FImpute v2.2 software [2].

### Accuracy of imputation

Accuracies of imputation were calculated using both genotype concordance rate and allelic R^2^ measures on a per-animal basis. The genotype concordance rate is calculated by comparing the imputed sequence genotype to the true sequence for the same animal (in the case the sequence calls from the 1000 Bull Genomes Project pipeline, as described by Daetwyler et al. [13]). Correct calls are scored as 1 while incorrect calls are scored as 0, even if they are partially correct (calling a heterozygote when the correct call is a homozygote or vice-versa).The genotype concordance rate is the average of these scores across all sites for a specific animal. Concordance rates were also measured for only sites with minor allele frequency (MAF) lower than 0.05. Allelic R^2^ was calculated as the squared correlation of the imputed sequence genotype on the true sequence.

### Using imputed sequences as reference

Increasing reference size by including imputed sequence genotypes in the reference population was explored by using Euclidean distances to select the animals that would benefit most from having a larger reference population, likely because of rarer haplotypes. Euclidean distances between each pair of animals were calculated based on the following formula:

$$ \sqrt{\sum \limits_i^n{\left({q}_i-{p}_i\right)}^2} $$

Where *q* and *p* are the genotype or haplotype states at locus *i,* for each of *n* loci for genotypes and each of *n* pseudo-genotypes when using haplotypes. Using Euclidean distance captures both the effects of relatedness between individuals, as well as allele frequencies. After Euclidean distances were calculated between each pair of animals, we determined which animals were not expected to be imputed well. Animals with little relation to the rest of the population were thought to be poorly imputed, and so animals with a mean or minimum Euclidean distance two or greater standard deviations above the mean were placed in the “poorly imputed” groups. This resulted in 90 and 121 animals for average and minimum Euclidean distance, respectively. The imputed sequence genotypes from animals with high degrees of relatedness were included in the reference population along with the rest of the population to impute the small group of animals predicted to be imputed with low accuracy based solely on average Euclidean distance. This same procedure was carried out again using only the minimum Euclidean distance. Animals with a high minimum Euclidean distance have no relative in the reference population or any animals that share a significant portion of the genome and are likely to benefit greatly from a larger reference population with a haplotype library more indicative of the haplotype frequencies in the overall population in order to maximize imputation accuracy.

### Predicting imputation accuracy

A predictive model was created for accuracy of imputation to sequence using only information available on the 50 K panel. First, to capture the degree to which each animal was related to the reference population for imputation. Euclidean distances were calculated using both 50 K genotypes, as well as haplotypes reconstructed using the PLINK algorithm, as described above, to determine which method predicted more efficiently the accuracy of imputation. A 5-fold random cross validation was used to predict imputation accuracy of each subgroup, using the other four groups to estimate the effect and significance of each term in the model. The effects tested for the model development were: breed, PLINK cluster based on both haplotypes and genotypes, ADMIXTURE cluster, maximum, minimum and average Euclidean distances for both genotype and haplotype, as well as number of parents and number of progeny in the sequenced reference population. The models were built based on backwards selection. In brief, after each model run, the least significant effect, based on model term *p*-value, was removed and the model was run again. Each time, the cross-validation was carried out, and terms were removed until the squared correlation between predicted imputation accuracy and true imputation accuracy decreased by more than 0.01 across the entire population. Once the final model was determined, the model was run one final time using all animals to get the optimal estimates for each linear model term for future imputation of animals to sequence using the 1000 Bull Genomes Project population as reference.

Additionally, a reduced model was investigated to ease the amount of computation and processing time. This was carried out in order to make a more practical model that could be used as a routine quality control step for removing poorly imputed sequences from subsequent analysis. This model used only Euclidean distances as calculated using 50 K genotypes, breed effects and pedigree information.

After parameter reduction, a simple linear model was constructed to predict imputation accuracy as follows:

$$ Accuracy={X}_{Breed}+{X}_{PlinkHapClust}+{\beta}_1\bullet EuclideanGeno Minimum+{\beta}_2\bullet EuclideanGeno Maximum+{\beta}_3\bullet EuclideanHaplo Average+{\beta}_4\bullet Number of Parents+ residual $$

Where X and β represent fixed categorical and fixed linear regression coefficient effects, respectively.

## Results

### Clustering methods

Each clustering method was restricted by the minimum number of animals per cluster (i.e. 150 individuals). Numbers of clusters, as well as minimum and maximum cluster size from each of the three clustering methodologies are shown in Table 1. ADMIXTURE had one fewer cluster than either method using PLINK, resulting in larger mean reference populations for imputation (278 individuals for ADMIXTURE method compared to 229 when using PLINK methodology). Clustering strategies were compared by looking at imputation accuracy after using clustered imputation from 50 K to 777 K masked genotype calls and then combining all animals together to impute to sequence. This was a reasonable method for testing clusters, as clustering should make a larger difference when imputing from a sparser low density panel.

**Table 1:** Summary statistics of Clusters from 3 different algorithms

Scenario	Number of clusters	Minimum cluster size	Maximum cluster size
ADMIXTURE	4	190	346
PLINK Genotype	5	168	280
PLINK Haplotype	5	172	278

The accuracy of genotype imputation from 50 K to full sequence in two steps for each of the three clustering methods as well as when using all animals as reference from 50 K to 777 K is presented in Table 2. The accuracy of imputation was measured by overall concordance rate, concordance rate for sites with MAF lower than 0.05, and R^2^. Table 2 also presents the average amount of computing time to impute each animal genotype/sequence. Clustering with ADMIXTURE as well as clustering using PLINK haplotypes was nearly equivalent and only two animals clustered differently across methods. Using PLINK haplotypes resulted in smaller populations and, consequently, slightly faster computing times.

**Table 2:** Accuracy of Imputation from 50 K to sequence, with either no clustering or different clustering algorithms used to determine reference population for imputation from 50 K to 777 K

Scenario	Overall R^2^	Time/Animal	Concordance	Concordance MAF < 0.05
All	0.819	0:03:47	0.934	0.974
ADMIXTURE	0.810	0:03:48	0.931	0.973
PLINK Genotype	0.807	0:03:37	0.930	0.973
PLINK Haplotype	0.810	0:03:44	0.931	0.973

### Imputation from 777 K to sequence

After imputation had been carried out from 50 K to 777 K, two methods were tested for imputation to sequence. First, using all animals as the reference population or secondly, using only animals within each cluster. One PLINK haplotype cluster was randomly chosen for this analysis due to the computational requirements of imputation to sequence. These animals were imputed either within cluster, split randomly into the same five subgroups that were used for imputation to 777 K, or by using results from imputation from step 1 where imputation was carried out on all animals. Genotypes on 777 K came from two separate sources: 1) those imputed from using all animals as reference from 50 K to 777 K or, 2) from using clustered reference populations. This resulted in four scenarios to be compared. The results of these comparisons based on genotype concordance, allelic R^2^, and computing time are presented in Table 3.

**Table 3:** Accuracy of imputation from 50 K to sequence, with either no clustering (ALL) or PLINK haplotype clustering (PLINKH) used to determine reference population for imputation from 777 K to sequence, and different reference populations (ALL or PLINKH) having been used for imputation from 50 K to sequence

Scenario		Overall R^2^	Time/Animal	Concordance	Concordance MAF < 0.05
50 K	777 K				
ALL	ALL	0.819	0:03:47	0.935	0.974
PLINKH	ALL	0.810	0:03:44	0.931	0.973
ALL	PLINKH	0.817	0:02:32	0.935	0.973
PLINKH	PLINKH	0.810	0:02:28	0.932	0.973

**Fig. 1:**
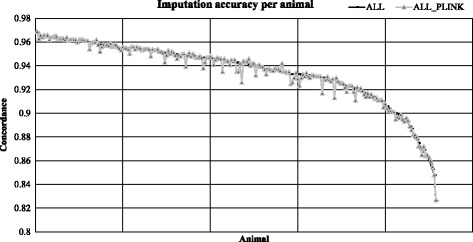
Imputation accuracy per animal from 777 K to sequence using all animals in reference, or using only animals within PLINK haplotype cluster as reference

Figure 1 shows imputation accuracy per animal when all animals were used as reference, as well as for PLINK haplotype clustering for 777 K to sequence imputation. For most animals clustering was a useful strategy to marginally increase imputation accuracy. However, for some specific individuals, clustering had a large negative effect on accuracy of imputation. This was further explored by breed, and average difference in imputation accuracy compared to using all animals in reference per breed by scenario was also examined, along with the proportions of animals that had improved accuracy of imputation per breed (Table 4). Accuracy of imputation from 50 K to 777 K was the largest factor in overall accuracy. In addition, using all animals for the first step was seen to provide the highest concordance rate and R^2^ values. There was little difference for imputation accuracy between scenarios from 777 K to sequence, but computation time was significantly reduced when using clusters or compared to using ADMIXTURE.

**Table 4:** Difference between concordance using PLINK clustering for one or two steps of imputation (directly from low density or low to medium to high) compared to using all animals in the reference population per breed and proportion of animals with improved accuracy for different imputation reference populations

Breed	PLINK (1 step) average change in accuracy after clustering	PLINK (1 step) Proportion of individuals in group with improved accuracy	PLINK (2 steps) average change in accuracy after clustering	PLINK (2 steps) Proportion of individuals in group with improved accuracy
Alberta Composite	0.035	0.714	0.004	0.714
Angus	0.005	0.727	0.004	0.727
Red Angus	0.000	0.000	0.00	0.400
Ayrshire	−0.001	0.800	−0.002	1.000
BeefBooster	0.005	0.000	0.022	0.000
Brown Swiss	0.021	0.917	0.020	0.917
Charolais	0.060	0.500	0.062	0.000
Gelbvieh	0.001	1.000	0.044	1.000
Guelph Composite	0.000	0.200	0.003	0.200
Hereford	0.000	0.429	0.002	0.286
Holstein	−0.007	0.500	−0.005	0.362
Red and White Holstein	0.001	0.250	0.005	0.000
Jersey	−0.014	0.833	−0.016	1.000
Limousin	0.054	0.667	0.063	0.500
Montbeliarde	−0.012	0.833	−0.003	1.000
Normande	−0.001	1.000	−0.002	1.000
Simmental	−0.028	0.500	−0.026	0.568

We also explored individual genome regions to ensure there were no areas significantly negatively impacted by clustering before imputation. These results can be seen in Fig. 3, which shows that there was no adverse effect.

### Using imputed sequences as reference

Accuracy of imputation for animals considered to be imputed poorly based on average or minimum Euclidean distances with the reference population were found to increase on average when imputed sequence genotypes were included in the reference population. Minimum Euclidean distance was a better predictor of poor imputation accuracy, as the group of individuals with minimum Euclidean distance more than one standard deviation greater than the mean had a very poor imputation accuracy when using the whole population of true sequences as reference (0.9272) (data not shown). When the reference population was expanded for this group by adding 130 imputed sequences (those animals in the same original imputation group with low minimum Euclidean distances), the accuracy of imputation remained unchanged, but improved for some animals. Of the 121 animals in the “poorly imputed” group, adding imputed sequences increased imputation accuracy for 89 animals, decreased accuracy in four individuals and made no difference in 28. The results of adding imputed sequences when using average Euclidean distance to predict poorly imputed individuals were also positive. An average increase of 0.001 was observed, and out of the 90 animals in the “poorly imputed” group, 61 had increases in accuracy, six sequences were more poorly imputed, and 23 had no change. Average change in accuracy by breed is included in Table 4 for breeds that had at least four animals in the “poorly imputed” population.

### Predicting imputation accuracy

A linear model using breed, ADMIXTURE cluster, PLINK genotype cluster, PLINK haplotype cluster, Euclidean minimum, maximum, and average distances for both genotypes and haplotypes, number of sequenced parents and number of sequenced progeny was initially tested. The initial model gave a correlation between predicted and actual genotype concordance of 0.8410 based on 5-fold cross validation.

**Fig. 2:**
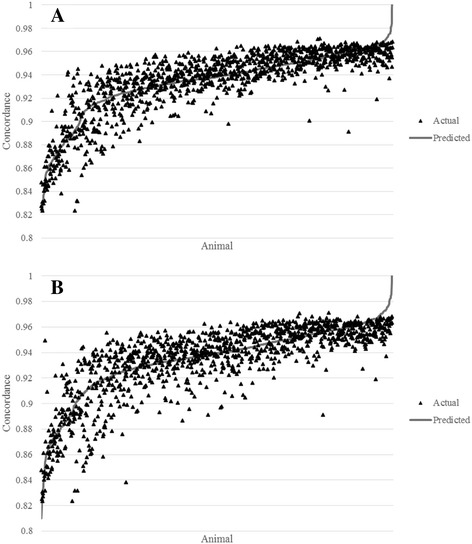
Actual vs. predicted accuracy for all animals using (**a**) the full prediction model or (**b**) a simple reduced prediction model

A reduced model was then tested after eliminating model terms with little or no effect. This model limited clustering steps to only using PLINK clustering based on haplotypes, and resulted in a correlation between predicted and actual imputation accuracy of 0.8710. Seven of the 10 worst individuals for actual imputation accuracy were predicted to be among the bottom 10 individuals, which is important for this model to be used as an exclusion criterion. Figure 2 shows the predicted versus actual imputation accuracy for all animals for the complete and reduced models.

A reduced model, containing only information from Euclidean genotypes was also tested. Breed, parent and progeny effects were once again tested in the model. It was found again that progeny effect was non-significant and it was removed. This model was less accurate than the full model as provided above, but maintained a correlation between actual and predicted imputation accuracy of 0.8330. In this case six out of the bottom 10 individuals for imputation accuracy were still predicted to be in the worst 10 imputed sequence genotypes. Furthermore, all 10 of the worst sequence genotypes were predicted to be in the lowest 25 for imputation accuracy (data not shown).

## Discussion

This study shows that reference population structure has a variable effect on imputation accuracy, which depends heavily on the breed to be imputed. Even with an imputation algorithm that uses population relationship information by searching for the longest possible matching haplotypes [2], including unrelated animals from different breeds in the reference population can negatively impact imputation accuracy, especially when reference population is small. However, with a small number of animals sequenced it is not possible to carry out within-breed imputation and expect to capture all or even a majority of the possible haplotypes needed for accurate imputation. This is especially true for breeds other than Holstein, Simmental, or Angus, who have fewer than 100 animals sequenced within breed to date [13]. In these cases, novel approaches to grouping animals needed to be explored and, as shown here, clustering of individuals for imputation to sequence can be effective for certain groups of animals. The results in this study were obtained from FImpute software, which its underlying algorithm essentially mimics a clustering approach based on haplotype similarity. The impact of clustering should also be investigated for other imputation methods in further studies. Genomic clustering methodologies have been shown to be effective at determining relationships between animals resulting from population divergence and admixing on a breed wide scale and this principle should apply well to an individuals’ genome also [14].

Clustering based on haplotypes rather than genotypes is a logical step for imputation accuracy, as sharing of haplotypes between reference and imputation population is the foundation of accurate imputation [15]. Haplotype blocks have been shown to be a strong indicator of breed relatedness. However, beef and dairy cattle breeds are not easily differentiated by using haplotype blocks [16]. Sharing of haplotypes examined by averaging across the genome, as was done based on the results from PLINK in this study, is a more logical step for imputation. All areas of the genome need to be treated and weighted equally when looking at imputation, to ensure there are no especially poorly imputed regions.

**Fig. 3:**
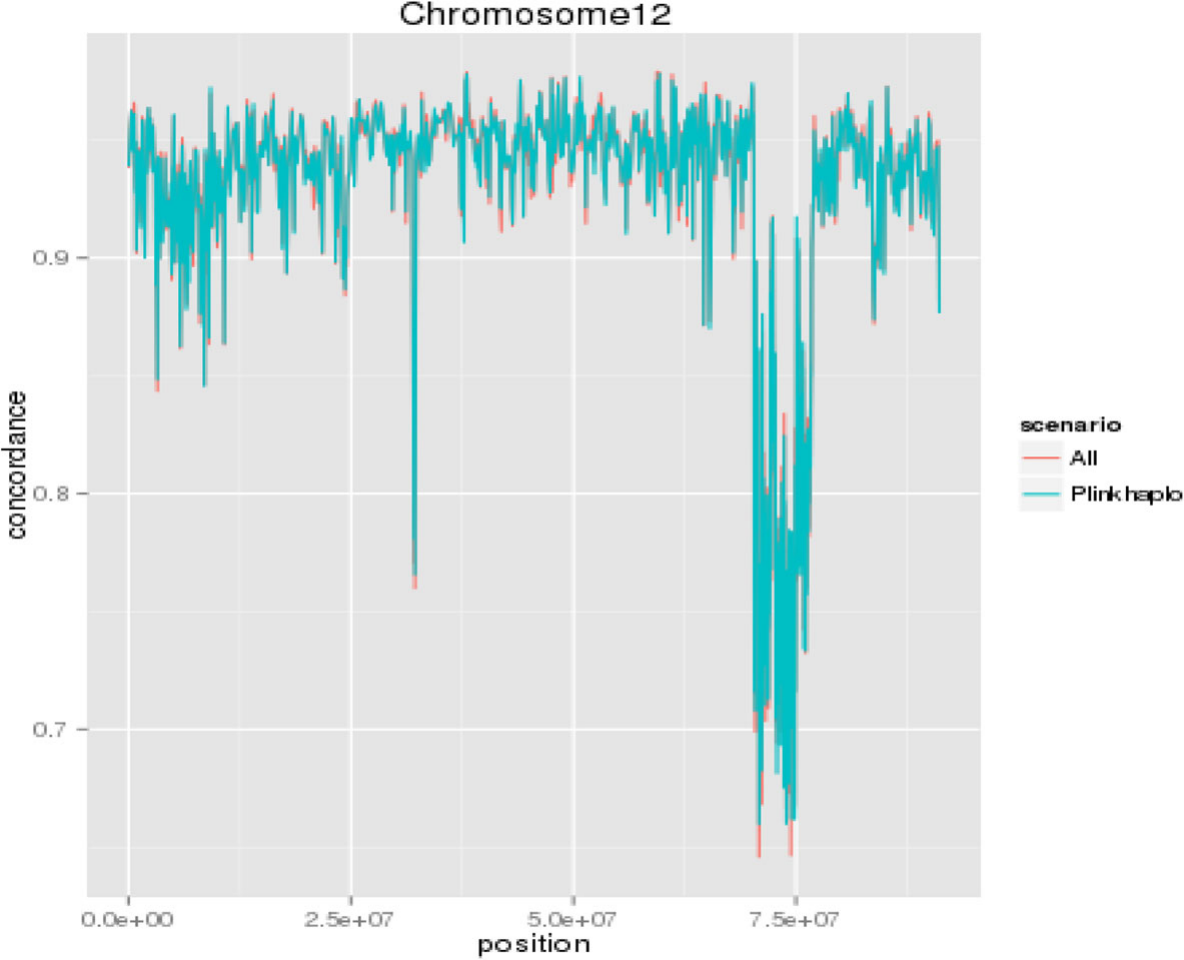
Imputation accuracy measured by genotype concordance by position on chromosome 12 when imputation was carried out using all animals as reference, or by clustering based on PLINK haplotypes

Figure 3 shows that when imputation is split into sub-populations by clustering, there are no adverse effects on any genomic region when comparing to using all animals as reference when using FImpute software for imputation. However, it also shows that poorly imputed regions cannot be significantly aided by altering the reference population structure. These regions that are poorly imputed may be due to longer haplotypes being needed and unavailable, or they may be reflective of recombination hot-spots, where a large number of haplotypes are present and, therefore, are poorly estimated by current imputation methods [17]. Poorly imputed regions could also be due to poorly annotated SNPs or greater levels of heterozygosity in these regions [18]. A solution to these problems is better SNP annotation. However, this is unlikely to be the problem at this point as the 50 K and 777 K bovine SNP chips have been tested by LD analysis and any misplaced SNPs have been relocated [19].

For long segments to be inaccurately imputed in this study due to poor annotation there would have to be entire sequence regions misplaced as accuracy was measured based on large windows of SNPs. This is unlikely to be the case as well, but could have some impact on accuracy of certain regions. The primary cause of poor accuracy in some regions is likely to be due to: 1) high haplotype diversity and low haplotype frequency; and, 2) poor concordance of commercial SNP markers and sequence calls in terms of minor allele frequency, leading to lower than expected levels of linkage between markers with short distances between them [5].

The only likely way to improve accuracy in regions of high heterozygosity and recombination is to increase the size of the reference population from the same breed. This allows for FImpute software, or other imputation algorithms, to more accurately choose shared haplotypes, based on length or shared haplotype probability, among individuals and choose haplotypes that are less likely to have undergone a recombination event between the reference and target individuals [2].

When using the FImpute algorithm, clustering reference populations for selection had no effect or a very small positive effect for most animals, as shown in Fig. 1, as well as on the proportion of individuals within each breed that had improved accuracy, as seen in Table 4. Other imputation algorithms that do not use decreasing window sizes, as FImpute software does, may stand to gain more from clustering before imputation. The reason for that is because FImpute software groups animals by relationships inherently by first looking at longest haplotypes and shrinking haplotype sizes progressively. However, there were a number of animals that had a significant decrease in imputation accuracy from a clustered reference population. These individuals were often crossbred or composite breed animals. Therefore, for some genomic regions haplotype frequencies and phases differ across breeds (i.e. different cluster); which can explain the decrease in accuracy. There were also certain animals that were clustered outside of their registered breed, and may be indicative of animals with a high proportion of sequence errors on sites shared between the sequence and 50 K SNP panel, or animals that have a registered breed that is not fully representative of their genome status. This may be due to varied rules for registering animals within a breed. Some breeds have closed herd books, and as such will share haplotypes primarily with animals only in their own breed (Dr. Stephen Miller, AgResearch, personal communication). These animals are likely to gain little from a clustering algorithm as short haplotypes that may be shared with other breeds are less common and thus less of a factor in imputation accuracy.

Animals from breeds with more lenient rules regarding breed status may gain more from clustering, as many shared haplotypes with other breeds may be needed for accurate imputation [7], and ensuring that only animals with relevant haplotypes are included in the reference population will reduce uncertainties where the wrong haplotype could be filled in. As presented in Table 4 clustering had a small or negative effect on most animals in the Angus and Hereford breeds, in which both breeds have closed herd books and, therefore, would stand to gain little from outside animals being included in the reference population. On the other hand, Simmental and Limousin animals, which come from a more lenient set of rules on purebred animal status, had larger proportions of animals that had increased imputation accuracy. Increasing accuracy of Limousin animals is a very positive result, as it has been a poorly imputed breed, even when compared to breeds of similar effective size and level of LD [4].

For many studies, there is a breed, or group of breeds, or crossbred animals that are the target population to be used for some subsequent analysis [20]. It follows logically, then, that improving imputation accuracy specifically for that group would be a research priority. Our findings show that there are ways to improve imputation accuracy for certain groups of animals, and tailoring the reference population for the target animals can have a positive effect on overall accuracy. The grouping/clustering of animals carried out in this study was done to try maximizing accuracy for all animals. However, there could be better ways to cluster animals to improve accuracy for a very specific group. Ventura et al. [21] reported that having animals in the reference population that well represented the breed composition of crossbred animals was essential to accurate imputation of crossbred beef cattle from 6 K to 50 K SNP chip panels. This study builds on that result showing that we can use genomic information to better estimate which animals should be grouped together for imputation or even genomic prediction purposes.

Using metrics such as Euclidean distances, it would be possible to only include animals in the reference population that are above a given threshold of relationship compared to the target population. It may be possible to further divide this group, if a more diverse set of animals was targeted. In this case, it would also be possible, unlike in this study design, for a sequenced individual to be in multiple reference populations if they shared haplotypes with more than one target imputation population. As it has been shown here, clustering with PLINK on haplotypes was the most effective tool for grouping animals. It is possible to calculate identical by state matrices for all animals in PLINK using either genotypes, or haplotypes as quasi-genotypes, which would be a useful tool for this type of intuitive clustering step before imputation [12].

It is well established that reference population size is one of the primary factors affecting the quality of imputation [22–24]. Although the FImpute imputation algorithm works based primarily on long haplotype sharing, in the absence of that, there is an inherent probabilistic nature to imputation. When many haplotypes are available to be chosen to fill in a specific set of SNPs for an animal or group of animals, often it is the most common haplotype that is filled in.

With small reference population size, the most common haplotype in the reference population may not be the most common in the entire population, especially not in the targeted population. Imputation to sequence creates a further problem in part due to a greater proportion of incorrect calls compared to SNP genotyping as well as a significantly lower average minor allele frequency [13]. To help solve this problem, we investigated the potential of adding imputed sequence genotypes to the reference population. If sequence genotypes that are known to be well imputed due to a high degree of relatedness to the original reference population are imputed first and added to the reference group, the haplotype library will become more representative of the total population. As the haplotype library better approximates the entire population, the probability of choosing the correct haplotype increases when the algorithm does not have long shared haplotypes to be used.

Euclidean distances were chosen as a simple metric to determine the average genomic relatedness between an individual and the reference population. At first it was thought that average Euclidean distance would be the ideal metric, as it captured the degree to which an individual was related to every animal in the reference. This should establish the degree of haplotype sharing between that individual and the reference [25]. However, it was observed that average Euclidean distance did not accurately predict which individuals were poorly imputed. This is likely due to having a large multi-breed training population so the average relatedness is not as much a metric of haplotype sharing, as it is of where an individual breed lies in the phylogenetic scope of the entire population. If that individual comes from a breed that has diverged further from most other breeds, it will have a much higher Euclidean distance. However, if there are other individuals of that breed in the reference population, those sequences may still be very well imputed. Conversely, a low average Euclidean distance may indicate an admixed or crossbred animal, that shares much of its genotype with many animals in the population, but for that same reason, is a difficult animal to impute due to a high degree of haplotype uncertainty in the imputation procedure.

Minimum Euclidean distance was chosen as an indicator of the closest relative in the reference population who would share a significant number of long haplotypes, and greatly improve imputation accuracy. This proved to be a much better indicator of imputation accuracy and seems to effectively select animals who will be well imputed. An increase in accuracy for individuals who were estimated to be poorly imputed was observed when imputed genotypes, selected by minimum Euclidean distance, were added to the reference population for imputation. Although this increase in accuracy is small, it is consistent across almost all animals and is a simple step that does not add significant computing time or require significant effort to achieve. There was not a single breed where the average imputation accuracy declined from adding imputed sequences into the reference population when predicted by minimum Euclidean distance.

There is potential to include easily predicted genotypes directly into imputation algorithms as an option, where each animal is imputed in order of minimum Euclidean distance, and then added to the reference population. The potential for this method lies primarily in sequence data where there is a greater number of calling errors and as yet a much smaller available reference population [13]. Once the sequenced population becomes larger there will be little need for this as true sequences are still much more accurate and representative of the true haplotype library of the population than imputed calls [26]. There is some risk that adding animals first who have low minimum Euclidean distances first will bias the haplotype frequencies in the reference population and lower imputation accuracy for less related animals, however this was not observed in this study.

Ensuring that only high quality sequences or genotypes are used for GWAS analysis, genomic selection or other genomic studies is crucial to limit the number of false positives and provide power to detect causal variants, as well as to increase the accuracy of genomic predictions. Rutkoski et al. [27] showed clearly that accuracy of genomic selection is directly affected by accuracy of imputation on a population wide level. It has also been shown that the power of GWAS studies increases as population size increases, but generally reaches an asymptote at a certain population size depending on the density of SNPs available and the relative risk per allele [28].

Animals are commonly removed from studies before imputation or GWAS due to a number of factors including MAF, call rate, and Hardy-Weinberg Equilibrium [17, 29, 30]. It follows then, that if imputation accuracy can be accurately predicted, it is also plausible to remove animals that are predicted to have imputation accuracy below a given threshold. Given the result that minimum Euclidean distance was a strong metric for imputation accuracy prediction, we attempted to use all data available to best predict imputation accuracy.

Figure 2 shows that the optimal model predicts imputation accuracy well, and more importantly there are few animals that are very poorly predicted. The animals that have very low imputation accuracy are generally predicted to perform poorly, which is the most important feature if this model is to be used as an exclusion criterion. Figure 2b demonstrates that a reduced model, although less accurate, will still accurately select the appropriate animals to remove from further analysis based on predicted imputation accuracy. For sequence analysis, removing poorly imputed animals could mean removing hundreds of thousands, or even millions of incorrect calls that could lead to spurious results, or decrease power to detect true associations between a causal variant and a trait of economic or practical significance [31]. This is especially relevant given the broad range of imputation accuracies seen in this study, ranging from as low as ~0.79 to ~0.97.

Even in breeds that are generally very well imputed such as Holstein there are some animals with imputation accuracies as low as 0.86 (data not shown). Being able to predict these individuals well is of utmost importance to subsequent analyses being as powerful and accurate as possible. It should be noted that the Holstein animal with the lowest imputation accuracy was predicted poorly in this study, with a predicted imputation accuracy of 0.91. However, this was the lowest predicted accuracy for a Holstein individual, and, therefore, in a purebred study, it would still be the most likely candidate to be removed.

Our findings indicate that genomic relatedness between an individual and the reference population is the key predictor of imputation accuracy on an individual basis and a linear model utilizing this information can be an effective tool to predict which animals will be poorly imputed. Reference population size has a large impact on imputation accuracy, and, consequently, as the reference population becomes larger, a model that relies on relationships between reference and imputation populations may have some diminished value. The reason for that is the smaller variance in the relationships between individuals in the population [2]. Although the specific parameters of the model may change given different reference sizes or breed compositions, it will likely hold that using genomic relationships or even haplotype relationships to predict imputation can be an effective quality control step to limit the inclusion of poorly imputed sequences or genotypes into a GWAS or genomic selection applications. Further investigation could also help to determine the best methods to add imputed genotypes or sequences to the reference population to try to impute animals with poor predicted imputation more accurately.

## Conclusions

Reference populations for imputation to NGS data can be more effectively chosen for both accuracy and computing time for certain groups of animals. Including imputed sequence genotypes in the reference population is a viable option to increase accuracy of poorly imputed individuals. Imputation accuracy can accurately be predicted to determine which animals will be imputed poorly, as a means of excluding those animals from subsequent analyses. Further studies on clustering crossbred animals for imputation purpose are required.

## Abbreviations

GWAS: Genome-Wide Association Studies
HD: High-density SNP chip panel
IBS: Identical by state
LD: Linkage disequilibrium
MAF: Minor allele frequency
NGS: Next Generation Sequencing
SNP: Single Nucleotide Polymorphisms

## References

[1] Calus MPL, Veerkamp RF, Mulder HA (2011). Imputation of missing single nucleotide polymorphism genotypes using a multivariate mixed model framework. J Anim Sci.

[2] Sargolzaei M, Chesnais J, Schenkel F (2014). A new approach for efficient genotype imputation using information from relatives. BMC Genomics.

[3] van Binsbergen R, Bink M, Calus MP, van Eeuwijk FA, Hayes BJ, Hulsegge I (2014). Accuracy of imputation to whole-genome sequence data in Holstein Friesian cattle. Genet Sel Evol.

[4] Li H, Sargolzaei M, Schenkel F (2014). Accuracy of whole-genome sequence genotype imputation in cattle breeds.

[5] Druet T, Macleod I, Hayes B (2014). Toward genomic prediction from whole-genome sequence data: impact of sequencing design on genotype imputation and accuracy of predictions. Heredity.

[6] Stachowicz K, Larmer S, Jamrozik J, Moore SS, Miller SP. Sequencing and genotyping for the whole genome selection in Canadian beef populations. Armidale: Association for the Advancement of Animal Breeding and Genetics; 2013. pp. 344-7.

[7] Larmer S, Sargolzaei M, Schenkel F (2014). Extent of linkage disequilibrium, consistency of gametic phase, and imputation accuracy within and across Canadian dairy breeds. J Dairy Sci.

[8] Ventura R, Larmer SG, Sullivan P, Miller SP, Schenkel FS (2016). Genomic clustering helps to improve prediction in a multi-breed. J Anim Sci.

[9] Steinthorsdottir V, Thorleifsson G, Sulem P, Helgason H, Grarup N, Sigurdsson A, Gudjonsson SA (2014). Identification of low-frequency and rare sequence variants associated with elevated or reduced risk of type 2 diabetes. Nat Genet.

[10] Hayes B (2014). Genomic prediction from whole genome sequence in livestock: the 1000 bull genomes project.

[11] Alexander DH, Novembre J, Lange K (2009). Fast model-based estimation of ancestry in unrelated individuals. Genome Res.

[12] Purcell S, Neale B, Todd-Brown K, Thomas L, Ferreira MA, Bender D (2007). PLINK: a tool set for whole-genome association and population-based linkage analyses. Am J Hum Genet.

[13] Daetwyler HD, Capitan A, Pausch H, Stothard P, Van Binsbergen R, Brøndum RF (2014). Whole-genome sequencing of 234 bulls facilitates mapping of monogenic and complex traits in cattle. Nat Genet.

[14] McKay SD, Schnabel RD, Murdoch BM, Matukumalli LK, Aerts J, Coppieters W, Moore SS (2008). An assessment of population structure in eight breeds of cattle using a whole genome SNP panel. BMC Genet.

[15] Li Y, Willer C, Sanna S, Abecasis G (2009). Genotype imputation. Annu Rev Genomics Hum Genet.

[16] Villa-Angulo R, Matukumalli LK, Gill CA, Choi J, Van Tassell CP, Grefenstette JJ (2009). High-resolution haplotype block structure in the cattle genome. BMC Genet.

[17] Hozé C, Fouilloux MN, Venot E, Guillaume F, Dassonneville R, Fritz S (2013). High-density marker imputation accuracy in sixteen French cattle breeds. Genet Sel Evol.

[18] Berry DP, McClure MC, Mullen MP (2014). Within- and across- breed imputation of high-density genotypes in dairy and beef cattle from medium- and low- density genotypes. J Anim Breed Genet.

[19] VanRaden P, Null D, Sargolzaei M, Wiggans G, Tooker M, Cole J (2013). Genomic imputation and evaluation using high-density Holstein genotypes. J Dairy Sci.

[20] Erbe M, Hayes BJ, Matukumalli LK, Goswami S, Bowman PJ, Reich CM (2012). Improving accuracy of genomic predictions within and between dairy cattle breeds with imputed high-density single nucleotide polymorphism panels. J Dairy Sci.

[21] Ventura R, Lu D, Schenkel F, Wang Z, Li C, Miller S (2014). Impact of reference population on accuracy of imputation from 6K to 50K single nucleotide polymorphism chips in purebred and crossbreed beef cattle. J Anim Sci.

[22] García-Ruiz A, Ruiz-Lopez FJ, Wiggans GR, Van Tassell CP, Montaldo HH (2015). Effect of reference population size and available ancestor genotypes on imputation of Mexican Holstein genotypes. J Dairy Sci.

[23] Marchini J, Howie B (2010). Genotype imputation for genome-wide association studies. Nat Rev Genet.

[24] International HapMap3 Consortium (2010). Integrating common and rare genetic variation in diverse human populations. Nature.

[25] Jakobsson M, Scholz SW, Scheet P, Gibbs JR, VanLiere JM, Fung HC (2008). Genotype, haplotype and copy-number variation in worldwide human populations. Nature.

[26] VanRaden PM, Sun C, O’Connell JR (2015). Fast imputation using medium or low-coverage sequence data. BMC Genet.

[27] Rutkoski JE, Poland J, Jannink JL, Sorrells ME (2013). Imputation of unordered markers and the impact on genomic selection accuracy. G3: Genes| Genomes| Genetics.

[28] Spencer CC, Su Z, Donnelly P, Marchini J (2009). Designing genome-wide association studies: sample size, power, imputation, and the choice of genotyping chip. PLoS Genet.

[29] Neibergs HL, Seabury CM, Taylor JF, Wang Z, Scraggs E, Schnabel RD (2013). Identification of loci associated with bovine respiratory disease in Holstein calves.

[30] Pryce JE, Johnston J, Hayes BJ, Sahana G, Weigel KA, McParland S (2014). Imputation of genotypes from low density (50,000 markers) to high density (700,000 markers) of cows from research herds in Europe, North America, and Australasia using 2 reference populations. J Dairy Sci.

[31] Hao K, Chudin E, McElwee J, Schadt EE (2009). Accuracy of genome-wide imputation of untyped markers and impacts on statistical power for association studies. BMC Genet.

